# On-item fixations during serial encoding do not affect spatial working memory

**DOI:** 10.3758/s13414-019-01786-5

**Published:** 2019-06-28

**Authors:** Stefan Czoschke, Sebastian Henschke, Elke B. Lange

**Affiliations:** 1 Max-Planck-Institute for Empirical Aesthetics, Grueneburgweg 14, 60322 Frankfurt, Germany; 2 Institute of Medical Psychology, Goethe University, Heinrich-Hoffmann-Strasse 10, 60528 Frankfurt, Germany

**Keywords:** Spatial working memory, Eye-movement control, Serial recall, Memory encoding, Corsi block task

## Abstract

Ample evidence suggests that there is overlap between the eye-movement system and spatial working memory. Such overlapping structures or capacities may result in interference on the one hand and beneficial support on the other. We investigated eye-movement control during encoding of verbal or spatial information, keeping the display the same between tasks. Saccades to to-be-encoded items were scarce during spatial encoding in comparison with verbal encoding. However, despite replicating this difference across different tasks (serial, free recall) and presentation modalities (simultaneous, sequential presentation), we found no relation between item fixations and memory performance—that is, no costs or benefits. Inducing a change from covert to overt encoding did not affect spatial memory performance as well. In contrast, regressive fixations on prior items, that were no longer on the screen, were associated with increased spatial memory performance. Regressions occurred mainly at the end of the encoding period and were targeted at the first presented item. Our results suggest a dissociation between two types of fixations that accompany serial spatial memory: On-item fixations are epiphenomenal; regressions indicate rehearsal or output preparation.

**Electronic supplementary material:**

The online version of this article (10.3758/s13414-019-01786-5) contains supplementary material, which is available to authorized users.

Eye-movements have been demonstrated to be highly related to visuospatial attention allocation (Chelazzi et al., 1995; Deubel & Schneider, 1996; Kowler, Anderson, Dosher, & Blaser, 1995; Shepherd, Findlay, & Hockey, 1986, see also the premotor theory of attention, e.g., D. T. Smith & Schenck, 2012). Just before saccadic eye movements are executed, attention is already shifted toward the saccadic goal (e.g., Deubel & Schneider, 1996). Furthermore, the eye-movement system is assumed to play a specific role for maintenance in visuospatial working memory (Baddeley, 1986; Belopolsky & Theeuwes, 2009a, 2009b; Morey, Mareva, Lelonkiewicz, & Chevalier, 2017; Pearson, Ball, & Smith, 2014; Postle, Idzikowski, Della-Sala, Logie, & Baddeley, 2006; Schut, Van der Stoep, Postma, & Van der Stigchel, 2017; Theeuwes, Olivers, & Chizk, 2005; Theeuwes, Van der Stigchel, & Olivers, 2006; Tremblay, Saint-Aubin, & Jalbert, 2006), as well as the other way around (Van der Stigchel & Hollingworth, 2018). That is, the constructs of visuospatial attention and visuospatial working memory are highly related to the eye-movement systems. In our study, we are particularly interested in the question of how eye-movement control is applied during the encoding of serial spatial information for memory recall.

A plethora of studies have been conducted to investigate the relation between eye-movement control and visuospatial working memory, and we will summarize the key results, reporting evidence for saccadic interference as well as rehearsal benefits. Because of the memory component, one important experimental set-up is to use a delayed-recall design. An overlap of structures predicts that saccades to nonmemorized locations result in interference with memory representations, whereas saccades to to-be-remembered locations should benefit performance, as they might qualify as rehearsal. In fact, research on the effect of eye movements to nonmemorized positions in the retention interval has presented evidence for saccadic interference with spatial memory (Hale, Myerson, Rhee, Weiss, & Abrams, 1996; Postle et al., 2006) and memory for shapes (Schut et al., 2017). Interestingly, this effect of interference is over and above the deleterious impact of covert spatial attention shifts (Lawrence, Myerson, & Abrams, 2004; Pearson & Sahraie, 2003). This suggests that the eye-movement system contributes a unique part of interference to spatial memory maintenance. In agreement with this interpretation, activated memory representations in the retention interval (RI) can also alter eye-movement control. Specifically, saccade trajectories deviated away from memorized locations (Theeuwes et al., 2005; Theeuwes et al., 2006) and saccadic latencies into the hemifield, in which a location had to be remembered, increased (Belopolsky & Theeuwes, 2009b). Given these results, it seemed unlikely, that the saccade system is used for rehearsal. Indeed, a study investigating eye movements in the delay period found rather low oculomotor activity (e.g., Pearson & Sahraie, 2003, Experiment 5). However, there is some evidence for eye movements as supportive mechanisms for rehearsal. For example, fixating target positions in the retention interval of a spatial serial-recall task showed a beneficial effect for fixation sequences that matched the serial presentation of items: memory performance increased (Tremblay et al., 2006). By manipulating open rehearsal activity experimentally via instructions, Godijn and Theeuwes (2012) demonstrated a benefit for saccades to the first three to-be-recalled locations in comparison with the control condition. Overtly targeting the last three digit positions, however, impaired performance for most of the accessed as well as not-accessed item positions, but produced no benefit for any. This result points toward a specific connection between eye movements and serial order. Comparing free viewing with a condition, where subjects were allowed to fixate one self-chosen position, revealed no difference between the conditions, even though the number of fixations differed dramatically (free: 14 vs. fixation: 1). That is, saccadic activity in general neither boosted nor impaired memory representations. One solution for the divergent results might be individual differences in preferences of saccadic control (Laeng & Teodorescu, 2002; Ridgeway, 2006). Some participants might choose more, and others less, saccadic activity.

Further evidence for oculomotor support of memory maintenance comes from studies outside the serial recall literature. For example, in the “looking-at-nothing” paradigm, it has been demonstrated that fixations on a blank position on a screen cue information associated with this position (Ferreira, Apel, & Henderson, 2008; Johansson & Johansson, 2014). In addition, fixation pattern in a stimulus-free delay period of a spatial change-detection task showed high similarity with eye movements during encoding (Olsen, Chiew, Buchsbaum, & Ryan, 2014), particularly for increased task difficulty (Wynn, Olsen, Binns, Buchsbaum, & Ryan, 2018). This indicates that eye movements during the retention interval might reanact encoding behavior. A match between reenactment and encoding behavior might support memory recall (Laeng & Teodorescu, 2002). However, there is also evidence against the reenactment/reinstatement hypothesis (Foulsham & Kingstone, 2013; Johansson, Holsanova, Dewhurst, & Holmqvist, 2012).

Important progress in understanding the role of eye-movement control in visuospatial working memory has been derived by studies using the eye abduction paradigm (Ball, Pearson, & Smith, 2013; Pearson et al., 2014), which allows disentangling the effects of oculomotor control, eye movements, and attention in a very controlled way. The abduction paradigm revealed selective impairment for spatial memory maintenance when stimuli are presented outside the oculomotor range (Pearson et al., 2014), but not for other visual features (Ball et al., 2013). Interestingly, using the eye abduction paradigm, it has also been demonstrated that the oculomotor control system plays an important role during the encoding phase of a spatial memory-span task as well (Pearson et al., 2014). However, the role of effective saccadic movements during the presentation phase of serial spatial memory tasks is still under debate (Lange & Engbert, 2013; Morey et al., 2017; Patt et al., 2014; Saint-Aubin, Tremblay, & Jalbert, 2007). Free viewing in a spatial serial recall task showed rather low numbers of fixations on to-be-remembered item locations (Lange & Engbert, 2013; Patt et al., 2014) as well as small saccadic amplitudes and long saccadic reaction times, whereas fixation probabilities on to-be-remembered verbal items were high (Lange & Engbert, 2013). Results are indicative of the active suppression of saccades during spatial and unimpeded execution during verbal memory encoding. Studies using distractor designs converge on those findings. Irrelevant concurrent saccades during encoding of a spatial memory task decreased spatial memory performance (Guérard & Tremblay, 2011; Guérard, Tremblay, & Saint-Aubin, 2009; Lange, Starzynski, & Engbert, 2012; Postle et al., 2006). This was also true when saccades were generated in a reflexive manner (Lange et al., 2012; Lawrence, Myerson, Oonk, & Abrams, 2001), or without visually presented saccadic goals (Postle et al., 2006), but not during postrotational nystagmus (Postle et al., 2006), which causes involuntary eye movements. Results strongly suggests that the loci of distractor interferences are processes involved in eye-movement control (Postle et al., 2006), not movements per se, similar to conclusions from the eye abduction paradigm. However, when manipulating eye-movement control by instructions, results are less clear. When participants had to trace upcoming stimuli, memory for spatial as well as verbal serial recall was impaired (Lange & Engbert, 2013), indicating general dual-task costs by forced-viewing instructions. On the contrary, Saint-Aubin et al. (2007), using a similar procedure, found a beneficial effect for forced item tracing compared with free viewing. This points to a general problem with forced viewing instructions: The affordances of the task might enforce adaptive behavior. In addition, costs based on the dependent variable (e.g., eye-movement control) are difficult to separate from dual-task costs.

To sum up, there appears to be overlap of the oculomotor system and spatial working memory. Investigating this connection has developed in two branches of research: The role of eye movements in spatial encoding and the role of eye movements in spatial memory maintenance. However, both are not independent, as is obvious for sequential encoding paradigms. The sequential encoding over a series of several items is not merely a matter of item encoding. Increasing serial positions requires subjects to encode upcoming items while simultaneously maintain an increasing number of prior items in memory within a common time frame. Therefore, overt fixation behavior in the encoding phase cannot be interpreted exclusively in terms of encoding demands or strategies, but interference might contribute to saccadic control as well as overt rehearsal processes, counteracting forgetting. To our knowledge, there is no study that has investigated these supposedly conflicting processes during the encoding phase of a spatial memory task.

## Experiment 1

Evidence on the role of on-item fixations during presentation in a spatial serial-recall task does not converge toward a common conclusion. Fixations might be beneficial (e.g., Saint-Aubin et al., 2007), saccades might be suppressed because of interference (Lange & Engbert, 2013; Patt et al., 2014), or eye behavior might be optimized to fit individual strategies (e.g., Laeng & Teodorescu, 2002). In addition, regressions after stimulus presentation have been investigated in the retention interval only (Godijn & Theeuwes, 2012; Morey et al., 2017; Tremblay et al., 2006). The potentially beneficial effect of rehearsal during the memory encoding phase has not been investigated so far in serial recall paradigms. We study both types of fixations (during and after presentation) separately as well as individual differences in eye-movement control.

We decided on a comparative design, in which features of visually presented stimuli had to be encoded into either the verbal or spatial domain. On each trial, participants saw a series of five spatially distinct bigrams and had to recall either the verbal content, the spatial positions, or both features. Importantly, participants were free to move their eyes, allowing us to measure natural viewing behavior. We chose two approaches to understand the behavioral consequences of eye movements. First, we related fixations toward on-screen items as well as fixations on previous item positions (i.e., regressions^1^) to memory performance in a correlative, observational account. It is currently unknown whether low-fixation tendencies during serial spatial encoding (e.g., Lange & Engbert, 2013) reflect systematic avoidance of item-targeting saccades, and second, whether regressions that are carried out during the encoding episode reflect maintenance processes. Both behaviors can be interpreted as strategic when they clearly improve memory performance. Second, based on our earlier study (Lange & Engbert, 2013), we expected low-fixation probabilities on bigrams in the spatial memory condition, but high-fixation probabilities in the verbal memory condition. To investigate whether these diverging oculomotoric behaviors are based on task-specific affordances, we added a critical third condition, in which subjects memorized both the verbal content as well as the spatial position of the stimulus (combined condition). We reasoned that, having to encode two different materials that, in isolation, elicit different preferred oculomotor behavior, introduces a conflict (i.e., making saccades toward the items for verbal encoding versus suppressing saccades toward the items for spatial encoding/maintenance). Importantly, memory accuracy can be analyzed separately for verbal and spatial performance in this combined condition, which will uncover how a change in fixation probabilities between the single tasks and the combined condition will affect memory accuracy. If participants apply a strategy with high-fixation probability (as in the verbal single task), and fixations are detrimental to spatial encoding, then the performance in the spatial task will dramatically decrease in relation to the spatial single-task condition. Alternatively, if participants choose a low-fixation strategy (as in the spatial single task), and if this strategy benefits spatial encoding but hinders verbal encoding, impairment of memory performance will be particularly strong for verbal recall in comparison with the verbal single task.

### Method

#### Participants

Thirty adults (20 females; ages 17–37 years; *M* = 24.13 years, *SD* = 4.35) participated in the experiment after giving written informed consent. All participants had normal or corrected-to-normal visual acuity. They were naïve to the purpose of the experiment and were paid for their participation (€10/hour). The experimental session lasted about 60 min.

#### Apparatus

Stimuli were presented on a 24-in. monitor (resolution: 1,920 × 1,080 pixels, refresh rate: 144 Hz). The experimental procedure was controlled by Python 2.5 and PsychoPy 1.8. We tracked the right eye with a sampling rate of 1000 Hz (EyeLink 1000, SR Research). A forehead and chin rest reduced head movements and was located 60 cm in front of the monitor. The experiment took place in a sound-attenuated booth, with the experimenter placed outside the booth but connected via an intercom.

#### Materials

**Fig. 1:**
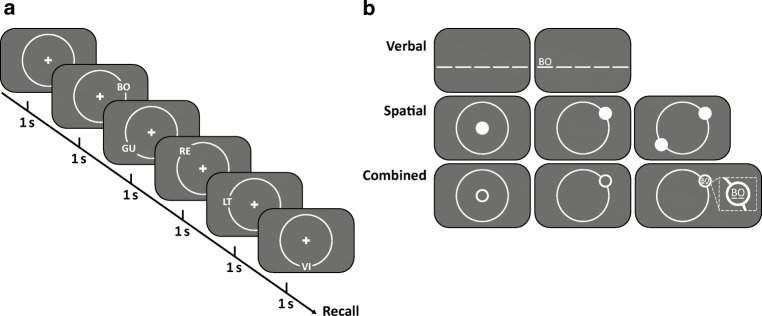
Scheme of item presentation (**a**) and recall (**b**) for the different conditions (verbal recall, spatial recall, combined recall) in Experiment 1. **a** After initial fixation, participants saw five bigrams sequentially presented with a rate of 1 item/s. Following the fifth item, recall began immediately. In the verbal (spatial) recall condition the five bigrams (bigram positions) had to be recalled in order of presentation. In the combined condition both features had to be recalled in alternation

Memory lists were composed of five bigrams. Items were constructed from two distinct letter pools. The first letter of each bigram was randomly drawn from [B, C, G, L, R, V], the second letter from [A, E, I, O, U, T] without replacement. The letter *T* was included in this second pool, to increase task difficulty, which had been pretested by a few pilot participants. This was important because we aimed at comparable task performances for the verbal and spatial task while keeping the list length equal (as this is required for the combined condition). The font color of the bigrams (letter height: 1° visual angle, bigram length: 1.5°) was white on a gray background (RGB: 128, 128, 128). Stimuli were shown on an isoeccentric, light-gray ring (RGB: 170, 170, 170) with a radius of 8° of visual angle (see Fig. 1). Item positions were randomly sampled on the circle without replacement from 20 equidistant positions (separated by 18 angular degrees on the circle or 2.5° of visual angle, and rotated by 7 angular degrees to avoid cardinal positions).

#### Design

Conditions (verbal, spatial, combined recall) were blocked, with two blocks per condition (six blocks in total). Serial order of conditions was balanced across participants. The first three blocks and the second three blocks comprised each condition once, respectively. Each block comprised 15 trials, with the first two trials being practice trials and excluded from data analysis, resulting in 26 trials per condition in total.

#### Procedure

The session started with a standard 9-point calibration of the EyeLink software. Participants initiated each trial by pressing the space bar (see Fig. 1 for a trial sequence). Each trial began with a fixation check, lasting 800 ms, which failed when the fixations deviated more than 1° visual angle from the centrally presented fixation cross. Calibration was repeated, when the fixation check failed twice or at the latest after five trials. Upon successful fixation check, the first item occurred. Each item remained on the screen for 1,000 ms, followed by the onset of the next item (see Fig. 1 for a trial sequence and the different recall procedures). After the fifth item, the recall display occurred without delay. Participants were instructed to report the items in order of presentation and to guess in case they did not remember; correction of a given answer was not possible. In the verbal task, recall was achieved by entering the bigrams via keyboard. In the spatial task, recall was achieved by moving the mouse pointer to the remembered positions and confirming each position by mouse click. In the combined task, recall was achieved by mouse-clicking on the remembered position and entering the respective bigram. When keeping list length equal, spatial recall usually results in lower task performance than verbal recall. We decided on first spatial then verbal recall, to motivate participants to keep track of the spatial task and not to ignore it.

#### Data treatment

##### Categorization of saccades

Eye-movement data were categorized into saccades and fixational eye movements, using the velocity-based algorithm from Engbert and Mergenthaler (2006; in our study Lambda = 10). Saccades with amplitudes shorter than 0.7° visual angle, or with a duration less than 10 ms, were ignored. The algorithm detected 24,373 saccades for all participants and trials, with 812 saccades per subject on average (range: 477–1,394).

##### Fixations

The time interval between the end of one saccade and the start of the next was defined as fixation. Note that this term is a simplification, as during these intervals eyes are still moving on a smaller scale (Engbert, 2006). We computed the position of the eyes during fixations by calculating the median of the *x* and *y* coordinates. Visualization of the gaze within a trial convinced us that the median was preferable over the mean, as outlier positions related to blink and noise made the mean measurement noisy. An item was defined as fixated if the median position during the fixational movement was located within a radius of 2° of visual angle from item center. Fixation probabilities express on how many instances item fixations occurred at all. We use the term for fixations on items as long as they are visible on the screen, in comparison with regression probabilities.

##### Regressions

We defined a fixation as regression when the fixation matched the position of an item that was presented earlier in the trial sequence than the current item. Note that earlier items were no longer visible on the screen; hence, regressions were memory based. Regression probability calculates how often at least one regression was made (instead of calculating how many regressions were made on average).

##### Performance accuracy

We followed a strict serial recall criteria—that is, items had to be recalled in presentation order. Bigrams were regarded correct if both letters were correctly entered. Spatial positions were regarded correct if the reported position deviated less than 2° of visual angle from the center of the correct item.

##### Data analysis

All reported analyses of variance (ANOVAs) and *t* tests were based on a repeated-measures design. An alpha level of .05 (two-tailed) was set for all frequentist statistical tests. However, to evaluate our data in terms of evidence for the null hypothesis, we added Bayes factors (BF_10_), that quantify the likelihood of the alternative hypothesis (H1) relative to the null hypothesis (H0), given the data. Thus, technically, a BF_10_ > 1 indicates evidence in favor of the H1, whereas a BF_10_ < 1 supports the H0. So, for example, a BF_10_ = 3.50 indicates that the data are 3.5 times more likely under the alternative hypothesis than under the null hypothesis. In accordance with Kass and Raftery (1995), we consider BFs between 1/3 and 3 as inconclusive evidence. Consequently, we treat BF_10_ > 3 as support for the H1, and BF_10_ < 0.33 as support for H0. For Bayesian ANOVAs (Rouder, Morey, Speckman, & Province, 2012), we report only the BF_10_ of the best model (i.e., the factor combination with the strongest evidence against the null model that includes only between-subjects variance), except if the evidence in favor of the best model compared with another predictor combination was weak (BF_10_ of the model comparison < 3). All data analyses were conducted with the statistics software JASP (Version 0.8.6.0; JASP Team, 2018) and the default settings of the Bayes Factor package (Morey & Rouder, 2015); that is, Bayesian ANOVAs were computed with a multivariate Cauchy prior with a fixed-effects scale factor of *r* = .5, and a random effects scale factor of *r* = 1. Bayesian paired *t* tests (Rouder, Speckman, Sun, Morey, & Iverson, 2009) were computed with a Cauchy prior, with a width of *r* = .707. Priors were centered on zero.

### Results

The Results section is structured by three questions: (1) Do fixations on to-be-remembered items during their presentation affect memory performance—in particular, do they impair spatial memory? (2) Do regressions affect memory performance—in particular, do they improve spatial memory? (3) Do eye-movements strategies reflect general processes or rather individual differences in behavior?

#### On-item fixations and their relation to spatial memory encoding

##### Fixation probabilities

**Fig. 2:**
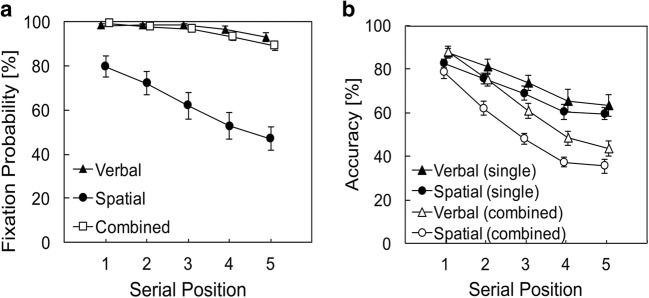
**a** Fixation probabilities on on-screen items for three different conditions: verbal immediate serial recall, spatial immediate serial recall, or both (combined condition) in Experiment 1. Fixation probabilities are markedly reduced for spatial encoding compared with the verbal and combined tasks that both show comparable ceiling effects. **b** Memory performance accuracy in the verbal and spatial task (single or combined), split for serial order. Items had to be recalled in presentation order (serial recall). Accuracy for spatial and verbal recall showed highly similar slopes in the single (filled symbols) and combined (unfilled symbols) conditions, respectively, suggesting domain-general costs associated with serial memory. The parallel slopes in the combined condition indicate that the high-fixation probabilities for combined encoding did not selectively interfere with spatial memory. Error bars depict between-subjects standard errors

Fixation probabilities on memory items during encoding were very high for the verbal-recall task, with only a slight decrease from the first (*M* = 98.21%, *SD* = 3.60) to the fifth (*M* = 93.08%, *SD* = 10.52) serial position (see Fig. 2a). For the spatial-recall task, fixation probabilities were markedly lower, with a strong, almost linear decline from the first (*M* = 79.74%, *SD* = 25.03) to the fifth (*M* = 47.05%, *SD* = 29.70) serial position.^2^ Crucially, the fixation pattern for the combined condition strongly converged toward the verbal condition (with *M* = 99.23%, *SD* = 1.56 for the first, to *M* = 89.23%, *SD* = 10.80 at the fifth serial position), and clearly differed from the observed fixation behavior in the spatial condition. Note that if participants chose to switch constantly between the fixation behavior of single verbal and single spatial encoding, serial position function in the combined task would be exactly placed in between the other two task’s functions. Note, too, that in the combined condition, spatial recall always preceded verbal recall, making it unlikely that the procedure itself biased participants toward concentrating on the verbal task.

The visual inspection of Fig. 2a was backed up by a two-factor ANOVA, with a significant main effect of condition (verbal, spatial, combined), *F*(1.03, 29.78) = 41.04, *p* < .001, η^2^ = .59 (Greenhouse–Geisser corrected), a main effect of serial position, *F*(2.51, 72.75) = 40.93, *p* < .001, η^2^ = .59 (Greenhouse–Geisser corrected), and an interaction, *F*(2.98, 86.50) = 17.94, *p* < .001, η^2^ = .38 (Greenhouse–Geisser corrected). Accordingly, the best model contained both factors and the interaction, BF_10_ = 8.47× 10^64^. Given the clear pattern of results, we want to report here one more detail only: When fitting verbal and combined in the two-factor ANOVA, the main effect of condition was significant, *F*(1, 29) = 6.35, *p* = .017, η^2^ = .18, but the interaction not, *F*(1.91, 55.36) = 2.61, *p* = .085, η^2^ = .08. Accordingly, the best model contained both factors, but no interaction (BF_10_ = 3.64 × 10^9^).

To clarify the quality of on-item saccadic suppression, we evaluated saccade numbers post hoc. In agreement with what has been shown by Lange and Engbert (2013), mean number of saccades did not differ for the verbal and spatial single tasks in Experiment 1 (*F* < 1). As the item fixation probability was lower in the spatial condition, but total saccade number was comparable between spatial and verbal, there was an increased investment of saccades onto nonitem positions and not a general inhibition of saccades.

##### Performance accuracy

Accuracy (see Fig. 2b) was highest in single verbal recall (*M* = 74.21%, *SD* = 20.41) followed closely by single spatial recall (*M* = 69.44%, *SD* = 11.36), and decreased performances in the dual-task situation (combined condition, denoted as subscript *c*, single as subscript *s*) for verbal_c_ (*M* = 63.23%, *SD* = 16.43), spatial_c_ recall (*M* = 52.31%, *SD* = 12.71). The three-factor ANOVA resulted in main effects of task domain (verbal, spatial), *F*(1, 29) = 7.47, *p* = .011, η^2^ = .21, task condition (single, combined), *F*(1, 29) = 81.44, *p* < .001, η^2^ = .74, and serial position, *F*(2.67, 77.54) = 199.21, *p* < .001, η^2^ = .87 (Greenhouse–Geisser corrected), and significant two-way interactions of task condition × serial position, *F*(4, 116) = 20.17, *p* < .001, η^2^ = .41, and task condition × task domain, *F*(1, 29) = 6.38, *p* = .017, η^2^ = .18. The two-way interaction of task domain × serial position and the three-way interaction were nonsignificant (both *F*s < 1). Accordingly, the best model contained all three factors and the interactions of task condition × serial position and task condition × task domain (BF_10_ = 9.68 × 10^99^).

##### Relation between fixation probability and performance accurac

First, we tested whether item fixations harm spatial memory by comparing the accuracy of spatial recall for fixated and nonfixated items in the single task situation only (low number of cases in the other conditions). However, since serial position is correlated with fixation probability and accuracy, we first calculated the mean accuracy score for each subject and serial position separately (including only serial positions that had values for both cases—fixation and nonfixation) and then averaged across all serial positions to ensure that each position had the same weight within the calculation. A paired *t* test revealed no significant difference of accuracy between fixated (*M* = 66.63%, *SD* = 14.84) and nonfixated items (*M* = 65.56%, *SD* = 18.54), *t*(29) = 0.42, *p* = .676, *d* = 0.08, BF_10_ = 0.21.

Second, we reasoned that, if item fixations were detrimental to the maintenance of spatial memoranda, the accuracy for spatial recall in the combined condition should be modulated by the degree of deviation from the ideal encoding strategy for spatial material (applied in the single task). That is, the more participants deviate in the combined task from their fixation behavior under mere spatial encoding demands, the greater the relative performance decrement should be for spatial memoranda in the combined condition. To test this prediction, we correlated the difference in fixation probability between spatial_s_ and the combined condition for each subject with the difference of accuracy between spatial_s_ and spatial_c_. The correlation, however, did not show any relationship between change in fixation probability and spatial recall accuracy, *r* = −0.165, *p* = .383, BF_10_ = 0.33. In other words, the change in item fixation behavior between the spatial_s_ and combined condition had no systematic influence on the spatial recall performance in the combined task. When participants were motivated to change their strategy from low-fixation probabilities (spatial_s_) to high-fixation probabilities (combined), memory for spatial information did not decrease in the combined condition. To sum, our manipulation to increase fixation probabilities during spatial encoding worked out. Importantly, the change in fixation behavior did not result in a systematic impairment for spatial serial recall.

#### Regressions and the relation to spatial memory encoding

##### Regression probabilities

**Fig. 3:**
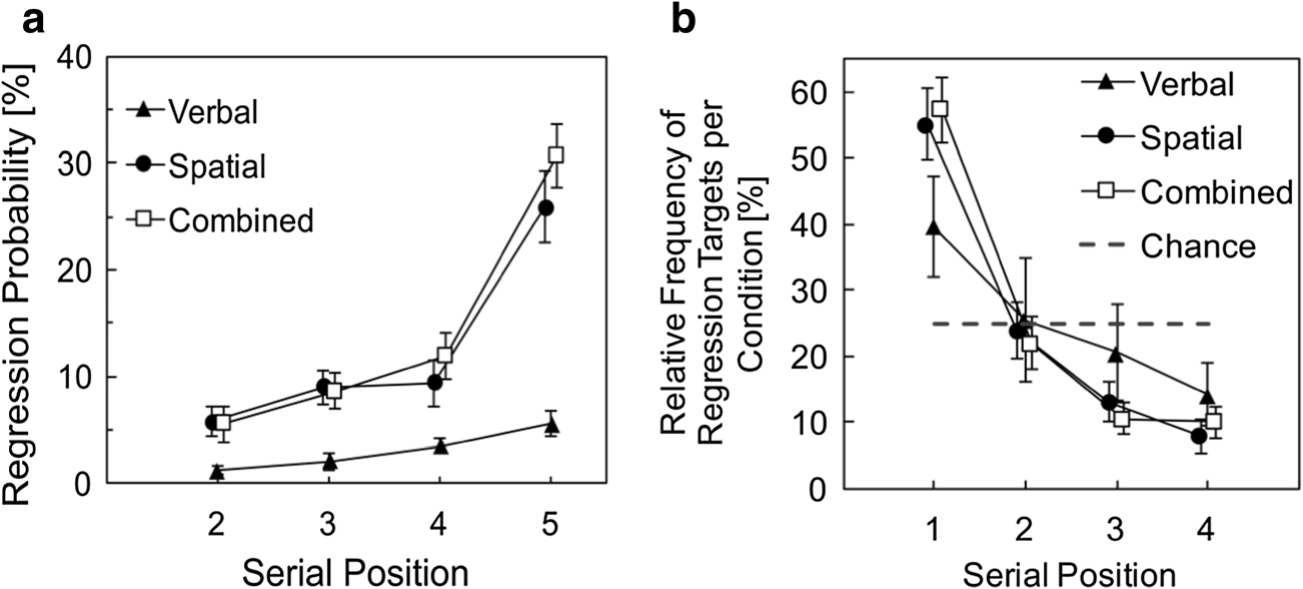
**a** Regression probabilities during the encoding phase in Experiment 1. Data are shown for the tasks (spatial, verbal, combined) and for Serial Positions 2 to 5, excluding the first serial position, because regressions were not defined. Tasks that involved spatial memory (spatial and combined recall) showed high regression probabilities, especially at the final list position. Regressions during mere verbal encoding were rare. Note that a slight linear increase as a function of serial position would also be expected by chance, since the visual area that is captured by previous stimuli increases with every list item of a trial. **b** Percentage of regression targets by serial position. Regressions predominantly targeted at the location where the first item occurred. Despite similar descriptive trends, only the spatial and combined condition differed from the chance model. Error bars depict between-subjects standard errors

Regression probabilities were similar in the spatial_s_ and combined conditions and more pronounced than in the verbal_s_ condition (see Fig. 3a). This interpretation was statistically supported by two related ANOVAs. The two-factor ANOVA resulted in significant main effects for condition (verbal, spatial, combined), *F*(2, 58) = 31.56, *p* < .001, η^2^ = .52, serial position, *F*(1.7, 49.314) = 42.13, *p* < .001, η^2^ = .59 (Greenhouse–Geisser corrected), and a significant interaction, *F*(3.49, 101.09) = 15.37, *p* < .001, η^2^ = .35 (Greenhouse–Geisser corrected). Accordingly, the best model included both factors and the interaction (BF_10_ = 2.08 × 10^41^). The main effect of condition and the interaction, however, were driven by the single verbal condition. When comparing regression probabilities for spatial_s_ and combined, there was no main effect of condition, *F*(1, 29) = 1.24, *p* = .275, η^2^ = .04, and no interaction, *F*(2, 57.89) = 1.85, *p* = .166, η^2^ = .06 (Greenhouse–Geisser corrected). Accordingly, the best model included only the factor serial position (BF_10_ = 8.28 × 10^24^).

As a complement, Fig. 3b depicts regression targets during presentation of the fifth item, which showed highest regression probabilities in Fig. 3a. Interestingly, there was a marked preference for regressions onto the first serial position. If regressions targeted accidentally on prior item locations, the frequency distribution should be uniform across all prior item positions. Note that such a preference for overtly revisiting the first serial position has been reported before for regressions during retention intervals (Godijn & Theeuwes, 2012) as well as during the encoding sequence (Lange & Engbert, 2013). To back up the interpretation of Fig. 3b, we calculated three one-factorial repeated-measures ANOVAs. The likelihood of being a regression target differed significantly between the serial positions in the spatial, *F*(1.92,53.66) = 21.32, *p* < .001, η^2^ = .43, BF_10_ = 7.34 × 10^10^ (Greenhouse–Geisser corrected), and the combined condition, *F*(1.85,53.54) = 28.56, *p* < .001, η^2^ = .50, BF_10_ = 1.58 × 10^14^ (Greenhouse–Geisser corrected), but there was no main effect of serial position in the verbal condition, *F*(3,48) = 1.61, *p* = .201, η^2^ = .09, BF_10_ = 0.68. However, evidence in favor of the H0 was very weak for the verbal condition. With a BF_10_ of 0.68, H0 is only 1.5 times (1/0.68) more likely than the H1, in our sample.

##### Relation between regressions and performance accuracy

Analogous to the analysis of fixation probabilities, we calculated the mean accuracy score for each subject and serial position separately (including only serial positions that had values for both cases) and then averaged across all serial positions to ensure that each position had the same weight within the calculation. Importantly, paired *t* tests revealed a significant performance benefit for regression targets (*M* = 81.63%, *SD* = 21.11) in comparison with items that did not become regression targets in the progression of a trial (*M* = 74.71%, *SD* = 13.43) in spatial_s_, *t*(28) = 2.27, *p* = .031. However, evidence for this performance benefit was very weak and rather inconclusive (BF_10_ = 1.78). There was no such performance difference for spatial_c_, *t*(29) = 0.80, *p* = .432, BF_10_ = 0.26. In addition, regressing onto the prior item’s position neither affected memory performance in verbal_s_, *t*(16) = 1.03, *p* = .320, BF_10_ = 0.39, nor in verbal_c_, *t*(29) = 0.27, *p* = .790, BF_10_ = 0.20, as expected.

Results indicate that regressions might be useful for remembering items that were regression targets (mainly the first item in the series). That is, in the single spatial task, there was some weak evidence for improved recall of regression targets in comparison with other items. However, verbal performance did not benefit from regressions at all, and neither did spatial memory in the combined condition, as expressed in the Bayesian analyses that favored the null hypothesis for these conditions.

#### Individual differences in oculomotor behavior during spatial memory encoding

**Fig. 4:**
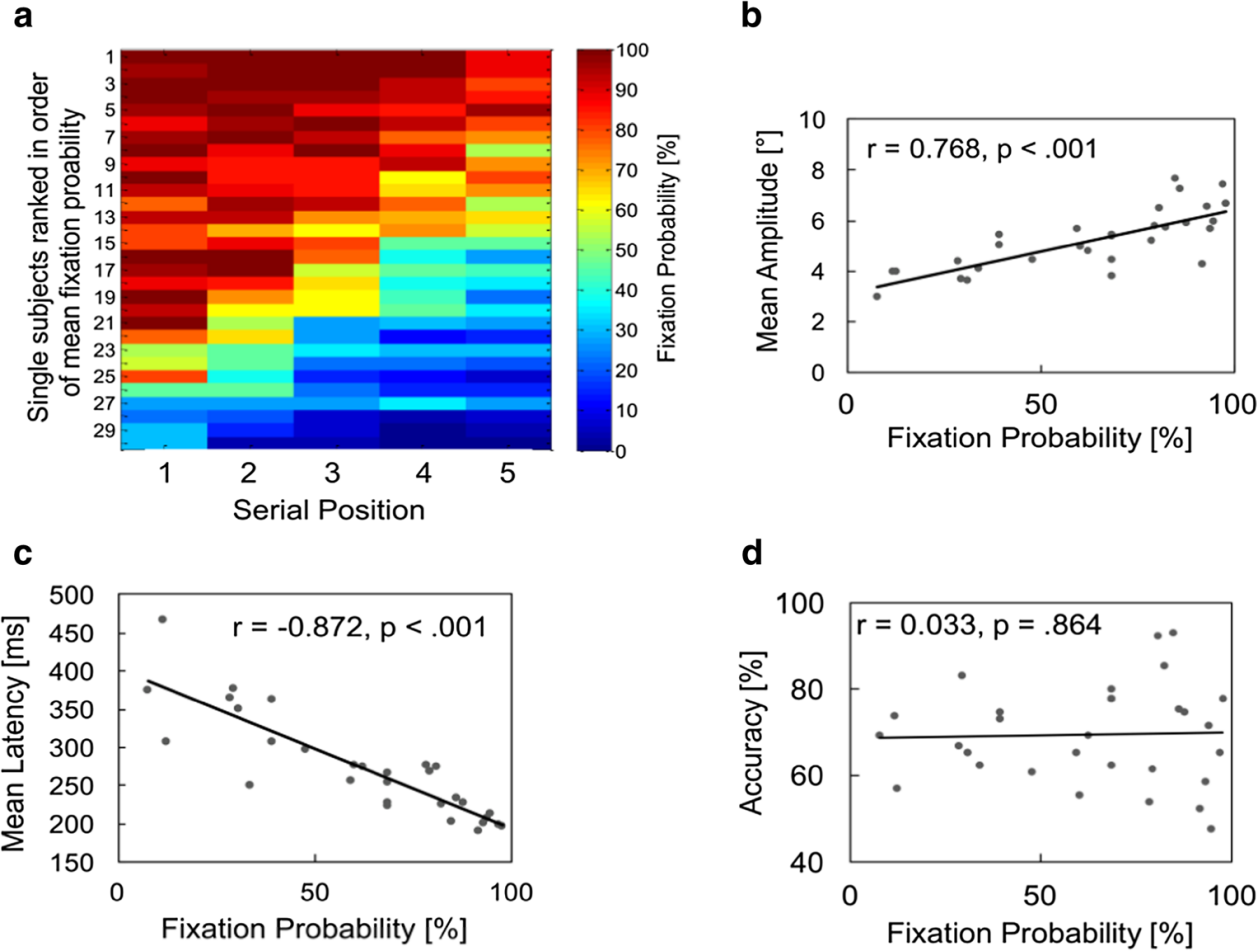
**a** Individual fixation probabilities onto items show large differences from consistently high (upper rows) to consistently low (lower rows) probabilities across serial positions. Participants with lower mean fixation probabilities show generally (**b**) smaller mean saccadic amplitude (BF10 = 2.67 × 104) and (**c**) higher saccade latencies, indicative of a general saccade suppression behavior (BF10 = 2.97 × 107). **d** This behavior is not functional on a general level—for example, fixation probability is not related to performance accuracy (BF10 = 0.230)

Figure 4a presents individual data of fixation probabilities for spatial encoding. As can be seen, there was a huge variability of individual encoding strategies, also depicted by the rather large error bars for spatial fixation probabilities in Fig. 2a. Interestingly, a subgroup of participants showed very high, others low, fixation probabilities across all serial positions. Participants used very different encoding strategies: from more overt to rather covert attention allocation. We can now ask further, whether low-fixation probabilities are related to other indicators of suppression, like higher saccadic latencies and smaller saccadic amplitudes (e.g., Ro, Pratt, & Rafal, 2000; Theeuwes et al., 2006). Indeed, Fig. 4b–c depict these correlations for spatial_s_, showing very systematic effects (see Supplementary Materials demonstrating no such systematic effects for the verbal and combined conditions). Figure 4d shows that suppressive eye movement behavior was not related to memory accuracy at all (also demonstrated earlier by relating accuracies with eye-movement behavior). That is, on the level of participants, systematic and strong differences in saccadic suppression occurred, but individual oculomotor activity did not result in accuracy differences.

### Discussion

We demonstrated saccadic suppression during spatial in comparison with verbal memory encoding, replicating earlier studies (Lange & Engbert, 2013; Patt et al., 2014). In addition, we observed a strong tendency for regressions under spatial encoding conditions at the final list position, which were mostly directed to the first location in the series. Whereas there was no evidence at all for saccadic suppression (to on-screen items) to be functional, we found weak evidence concerning the functional role of regressions (to items presented earlier in the series) in spatial memory maintenance. Importantly, we included also a condition, in which both contents (bigrams and their positions) had to be recalled. This manipulation was expected to result in high-fixation probabilities, and thereby would pose a challenge on spatial encoding, if on-item fixations during presentation were detrimental. In addition, regression probabilities in the combined task should match the spatial single task. Indeed, high-fixation and regression probabilities occurred in the combined task. But whereas regression probabilities related to spatial memory performance as predicted, fixation probabilities did not. Results on individual mean fixation probabilities suggest that participants differed on eye-movement control during encoding. Some used more overt visual attention allocation, and others applied systematic suppression of saccades. These specific behaviors did not relate to memory performance in general and also not to an individual optimization of encoding processes. Saccadic suppression does not indicate interference during spatial encoding, but it reflects differential applications of overt or covert attention allocation.

Regressions were mainly placed onto the first list item. Our results on regression targets converge nicely with existing WM models, suggesting that order information arises from coding the serial position of an item relative to the start of the memory list (e.g., Henson, 1998). The results are also compatible with the assumption, that encoding strength should be highest for the first item in a list, (e.g., Brown, Neath, & Chater, 2007; Lewandowsky & Murdock, 1989; Page & Norris, 1998), and the gaze is either supporting this or driven by this. Even though research on articulatory rehearsal processes indicates that the beginning of a list is rehearsed (e.g., Tan & Ward, 2008), we found high regression probabilities only in spatial but not in verbal recall. This points to a specific association between regressions and spatial memory. Regressions indicate maintenance processes, as the regression target, by definition, is no longer present on the screen and, consequently, new information about the item cannot be sampled. However, there was a sharp increase of regressions during presentation of the last item. This increase additionally indicates output preparation, as after the last item, presentation recall started immediately.

Arguably, the present dissociation for fixations and regression in two task domains (verbal, spatial) calls for the statistical test of an interaction—for example, a 2 (item fixated or not) × 2 (item regressed or not) ANOVA. However, the low number of cases for regressions as well as for nonfixated items (particularly in the verbal task) provide the difficulty to estimate sensible means to fit into an ANOVA. In addition, such an analysis should ideally include serial position as a confounding factor, which potentiates the problem of a low number of cases.

The serial position curves for memory accuracies are interesting for several reasons. First, the shape of the serial position curves matched for verbal_s_ and spatial_s_ serial recall, but differed only in intercept. This result is in line with the assumption that serial order memory is based on domain-general processes (Ward, Avons, & Melling, 2005). Second, the combined task situation had a detrimental effect on the slope of the serial position curve, which was third, again similar for verbal_c_ and spatial_c_ serial recall. The steepened slope eventually mirrors the fact that, due to the dual-task situation, encoding strength (e.g., Page & Norris, 1998) was diminished in the dual-task situation, or maintenance processes were hampered (e.g., rehearsal; Page & Norris, 1998), or there was less time for maintenance (e.g., refreshing; Barrouillet, Bernardin, & Camos, 2004), or recall for individual items was increasingly delayed (e.g., Brown et al., 2007). Whatever mechanisms were at play, the parallel slopes indicate that those mechanisms were likely domain-general and contributed to both features (verbal and spatial) to the same extend. Interestingly, parallel slopes make it unlikely that the high-fixation probability in the combined task hampered proper spatial memory encoding in a domain-specific way.

It is important to note that the memory decrements that we observed in the combined condition are well explained by the increased effort to encode two features per serial position (Langerock, Vergauwe, & Barrouillet, 2014). And it has been shown previously that spatial memory suffers more than verbal when combining both (Morey & Miron, 2016). Thus, our result of a stronger decrease for spatial in comparison with verbal recall by the combined condition does not reflect a stronger effect of the change in fixation behavior for spatial in comparison with verbal. The crucial analysis has to directly relate the spatial performance decrease to a change in eye-movement control. This is exactly what we did. We demonstrated a huge range of interindividual different eye-movement strategies during encoding. We compared fixation behavior between the spatial single and the spatial combined task. The more suppression of saccadic activity had to be reduced (from single to combined), the stronger memory impairment should be. However, this was not the case. There was no relation between a change in eye-movement behavior (from spatial single to combined) and a change on spatial memory performance.

Whereas fixations on to-be-encoded items during their presentation do not interfere with spatial memory and also do not play a functional role for spatial memory encoding, regressive eye movement acts as a maintenance process to support the beginning of the memory list, particularly.

## Experiment 2

The role of eye movements for memory encoding and maintenance has been discussed in a variety of task designs. We now ask whether our findings from Experiment 1 are specific for tasks including serial-order memory. To do so, we compared five different settings: Serial, free, and cued recall for sequential presentation, and serial and free recall for simultaneous presentation of the stimuli. Free and cued recall do not require participants to encode serial order. There is evidence showing that eye-movement behavior might be particularly related to serial-order memory (Tremblay et al., 2006). Whereas free recall with serial presentation usually shows some encoding in presentation order (Bhatarah, Ward, & Tan, 2008; Cortis, Dent, Kennett, & Ward, 2015; Grenfell-Essam, Ward, & Tan, 2017; Howard & Kahana, 1999), in cued (verbal) recall, items are less likely rehearsed in series (e.g., Henson, Hartley, Burgess, Hitch, & Flude, 2003). Serial position curves, indicative for serial order, have been demonstrated to differ for all three tasks (e.g., Murdock, 1968a, 1968b). But we included cued recall for another reason: In our task, the cue of which feature (verbal or spatial) had to be recalled was given after list presentation by the other then the to-be-recalled feature (spatial or verbal). This makes the task similar to our combined condition in Experiment 1, as both features had to be encoded. We expected fixation probabilities to be again very high, because spatial as well as verbal information had to be encoded for later cued recall. This would enable us to repeat the key analyses from Experiment 1, aiming at replication. The planned change in oculomotor behavior from low-fixation (free or serial recall) to high-fixation probabilities (cued spatial recall) should not relate to performance differences, indicating again that fixation behavior has no negative or positive consequences for memory encoding. In addition, regression probabilities should be higher in spatial than verbal tasks, and they should again be functional for serial recall. To the extent that regressions indicate maintenance in serial-order memory, regressions should be low in cued recall. Accordingly, the beneficial effect of regressions should replicate for serial recall and be attenuated in free recall. Low-regression probabilities in cued recall might forestall relating their occurrence to performance.

We included two more conditions with simultaneous presentation: free and cued recall. During sequential presentation, each upcoming item attracts attention (e.g., Yantis & Jonides, 1984) and the gaze due to singleton pop out (e.g., Kramer, Hahn, Irwin, & Theeuwes, 1999; Theeuwes, Kramer, Hahn, Irwin, & Zelinsky, 1999). A suppression effect might then be shadowed because oculomotor control might be driven by gaze capture, or boosted by inhibition of return (IOR), similarly to attentional capture (Fecteau & Munoz, 2006). In these cases, the simultaneous presentation might qualify as a baseline. If suppression is characteristic for spatial encoding in general, this effect should appear and even be more pronounced in the simultaneous presentation. The expected high-fixation probabilities during cued but not free spatial recall will again provide the possibility to replicate the nonexisting relation between a change of fixation behavior and recall accuracy.

### Method

We collected the data for this experiment together with memory tasks for colors, using the same serial, free and cued recall tasks. However, due to a programming error, results on color memory cannot be interpreted and thus are not reported. Data of the color task were collected in a different session (serial order counterbalanced, sessions at least 24 hours apart).

#### Participants

Thirty adults (21 females; ages 18–35 years; *M* = 24.12 years, *SD* = 4.69) participated in the experiment. The experimental session lasted no longer than 70 min.

#### Material

Verbal memory lists were composed of five bigrams, analogous to Experiment 1, with few changes. The letter pool of the first letter was [B, C, G, L, R] and of the second was [A, E, I, O, U]. We reduced item positions from 20 options to 12 equidistant positions on the circle (separated by 30 angular degree on the circle or 4.1° of visual angle, and rotated by 7 angular degree to avoid cardinal positions).

#### Design

The experiment comprised 10 conditions. Recall procedure (serial vs. free vs. cued) × exposition type (sequential vs. simultaneous) × recall feature (bigram vs. spatial position). Note that for simultaneous item exposition, serial recall is impossible, and hence the design comprised 10 and not 12 conditions. Conditions were blocked. Except for the cued recall, all conditions comprised one block of 22 trials (with the first two trials being practice trials and excluded from data analysis). Each cued recall condition comprised two blocks of 22 trials each (thus 44 trials in total; the first two trials of each block were practice trials). Within each session, serial order of conditions was balanced by a Latin square Williams design for avoiding first-order carryover effects (Williams, 1949).

#### Procedure

**Fig. 5:**
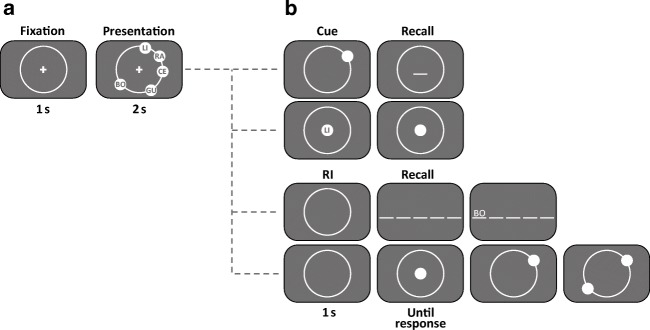
Example of simultaneous item presentation (**a**) and the recall conditions (**b**) in Experiment 2. **a** After initial central fixation, five bigrams were presented simultaneously for 2 s or sequentially for 5 s (1 item/s, not depicted). The sequential presentation procedure was identical to Experiment 1. Experiment 2 comprised six different recall conditions. In cued verbal recall (**b**, first row), a spatial cue appeared after item presentation was finished that indicated the position of the target bigram. In cued spatial recall (second row) a verbal cue appeared centrally that indicated the bigram of the target position. In serial and free verbal (third row) and spatial (fourth row) recall blank screen appeared followed by a whole report procedure. Note that the simultaneous presentation condition comprised only cued and free recall tasks, as serial order recall is impossible

The procedure was identical to Experiment 1, except for the following differences (see Fig. 5 for a trial sequence): After the fixation check, the five items were presented either in the same sequential manner as in Experiment 1, or for 2 s simultaneously. In the cued recall condition, the cue appeared immediately after item exposition and lasted 1 s, followed by the recall display. Here, the nonrecall feature served as the retrieval cue. In cued verbal recall, a light-gray disc appeared at one of the item positions. Subjects had to enter the bigram that was presented at that position via keyboard. In cued spatial recall, a bigram appeared at screen center. Subjects had to click the spatial position where the stimulus had appeared in the trial. For free and serial recall, a 1-s blank screen followed item exposition to match delayed recall of the cued recall condition. The recall procedures of free and serial recall were identical to the recall in the "single" conditions of Experiment 1.

Regarding all other aspects (consent, instructions, apparatus), Experiment 2 matched Experiment 1.

### Results

#### On-item fixations and their relation to spatial memory encoding

##### Mean fixation probabilities

**Fig. 6:**
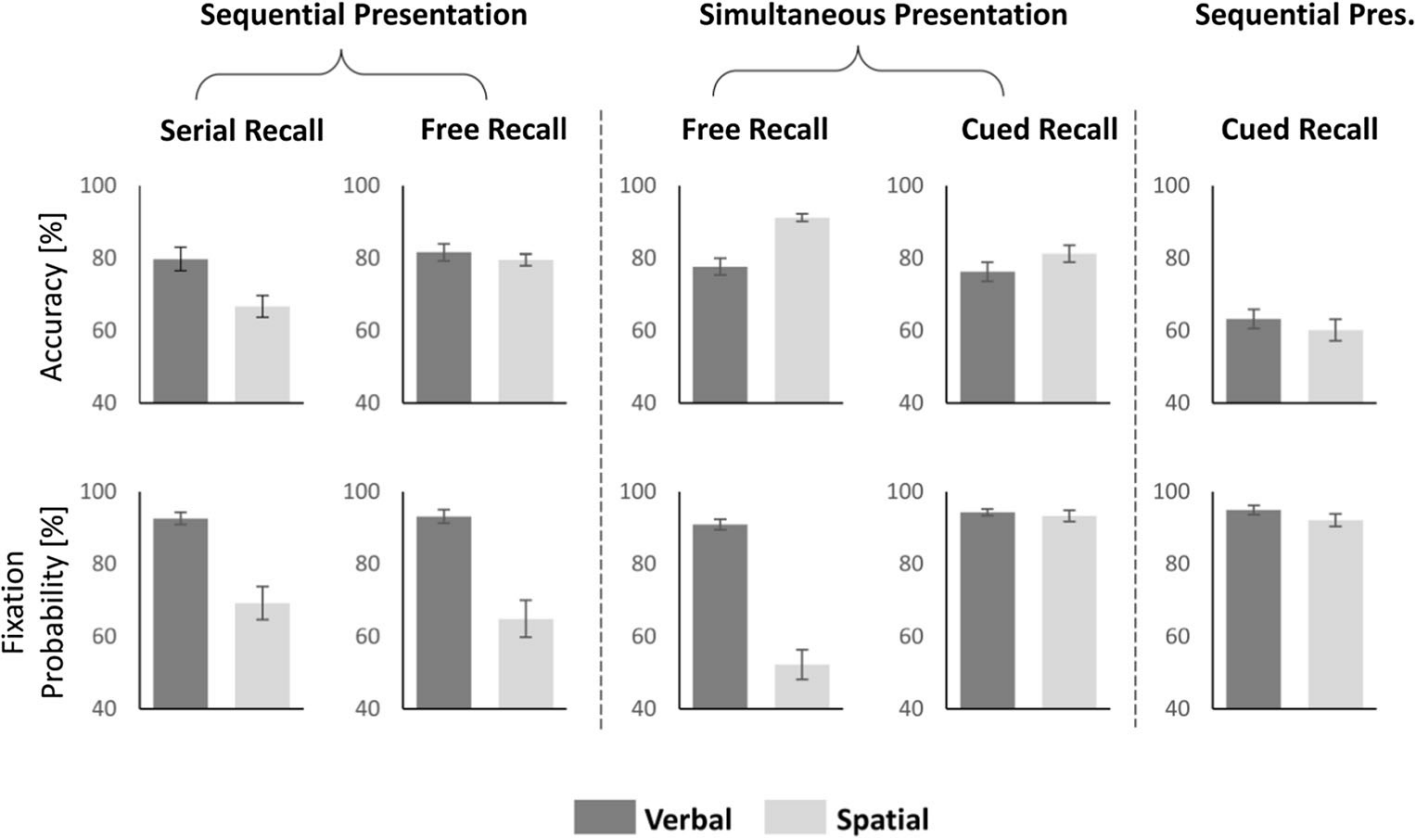
Differences in accuracies (upper panel) and mean fixation probabilities (lower panel) for different tasks (free, serial, cued recall), task domains (verbal, spatial) and presentation conditions (sequential, simultaneous). Error bars depict between-subjects standard errors

To simplify the Results section, we will no longer report serial-position effects, which mirrored what has been demonstrated already (e.g. diverging serial-position functions for fixation probabilities of verbal and spatial encoding in free and serial recall). Figure 6 depicts the mean accuracies and fixation probabilities for verbal and spatial encoding in five task conditions. It is clear from Fig. 6 (lower panel), that we replicated the suppression effect as expected: Fixation probabilities were lower during spatial than during verbal encoding in serial recall (sequential presentation) and both free recall conditions (sequential and simultaneous presentation). In addition, we again succeeded in our gaze manipulation using cued recall: Fixation probabilities were high in cued recall (above 92% in all conditions), much higher than during free and serial recall. There was behaviorally a small difference (verbal: *M* = 94.90%, *SD* = 7.00 spatial: *M* = 92.00, *SD* = 9.80) between domains in cued recall with sequential presentation, *t*(29) = 2.29, *p* = .029, *d* = 0.42, BF_10_ = 1.84, which we will not elaborate on, because the point was to create high-fixation probabilities in a spatial memory task. Fixation probabilities did not differ with simultaneous presentation (verbal: *M* = 94.30%, *SD* = 5.10 spatial: *M* = 93.20, *SD* = 8.60) , *t*(29) = 0.92, *p* = .367, *d* = 0.17, BF_10_ = 0.29.

As results are so clear cut and to limit the Results section to the important tests, we refrain from reporting comprehensive statistical tests on the saccadic suppression effect. But two tests might be worth mentioning: First, we report the two-factor ANOVA for fixation probabilities with the main effect of task domain (verbal, spatial) and task condition (free, serial recall and sequential presentation; see the two left-most subplots in Fig. 6, lower panel). This two-factor ANOVA resulted in a main effect of task domain, *F*(1, 29) = 42.35, *p* < .001, η^2^ = 0.59, which did not interact with condition, *F*(1, 29) = 1.40, *p* = .246, η^2^ = 0.05. The main effect of condition was not significant, *F*(1, 29) = 0.77, *p* = .386, η^2^ = 0.03. Accordingly, the best model included only the factor task domain (BF_10_ = 3.27 × 10^11^). That is, irrespective of the affordance to encode serial positions or not, saccades to item positions were suppressed during spatial memory encoding.

Second, we compared fixation probabilities during the encoding of a free recall task, presenting items sequentially or simultaneously (see Fig. 6, lower panel, second and third subplot from the left). Sequential presentation might have boosted fixation probabilities due to attentional capture. The two-factor ANOVA with task domain (verbal, spatial) and presentation mode (sequential, simultaneous) as factors, revealed a main effect of task domain, *F*(1, 29) = 88.60, *p* < .001, η^2^ = 0.75, a main effect of presentation mode, *F*(1, 29) = 6.88, *p* = .014, η^2^ = 0.19, and a tendency of an interaction, *F*(1, 29) = 4.29, *p* = .047, η^2^ = 0.13. The best model included the factors task domain and presentation mode (BF_10_ = 1.13 × 10^16^). However, the second-best model, which included the interaction, was comparable (BF_10_ = 1.02 × 10^16^). Results are a little weak regarding the interaction. However, the main effect of presentation mode confirms that fixation probabilities were overall higher during sequential presentation. This is best explained by capture.

Simultaneous presentation can be regarded as a baseline measure for the suppression effect. Fixation probabilities during the simultaneous presentation mirror rather endogenous allocation of attention. Here, the suppression effect occurred as well and was eventually stronger. The suppression effect was then replicated across tasks differing in serial order requirements and presentation modalities (simultaneous, sequential). That is, this effect is solid and highly replicable.

##### Relation between fixation probability and performance accuracy

It is already clear from Fig. 6, eyeballing potential relations between fixation probabilities and accuracies, that there cannot be a strong relation between those simple measures. To further support this conclusion, we analyzed the relation analogue to Experiment 1. Comparisons for performance between fixated and nonfixated items were carried out for cases in which fixation probabilities were not at the ceiling (fixation probability < 90%). For simplification of the Results section, Table 1 reports *t* statistics and BF_10_, replicating null-effects from Experiment 1 throughout.

**Table 1.** Summary statistics for comparisons of spatial memory performance accuracy between fixated and nonfixated items in Experiment 2

Presentation	Condition	*t*	*df*	*p*	Cohen’s *d*	BF_10_
Sequential	Serial	0.960	27	.346	0.181	0.305
Sequential	Free	0.882	28	.385	0.164	0.282
Simultaneous	Free	−1.145	29	.262	−0.209	0.353

*Note.* Statistics for the *t* test, comparing spatial task performance between fixated and nonfixated items in different presentation conditions (sequential, simultaneous) and task conditions (serial recall, free recall). We excluded comparisons for conditions with fixation probabilities > 90% (e.g., verbal tasks), because the small number of nonfixated items made the tests unreliable. We report *t*, df, *p*, a measure for effect size (Cohen’s *d*), and Bayes factor (BF), with BF10 > 1 indicating evidence in favor of H1 and BF10 < 1 supportive evidence for H0

Following the argumentation of Experiment 1, we also analyzed the relation between a change in spatial fixation behavior from serial to cued recall in the sequential presentation condition, and from free to cued in the sequential as well as in the simultaneous condition. Again, the change in oculomotor behavior did not correlate with the accuracy change. Table 2 report statistics on these correlations. Importantly, forcing participants to change their fixation behavior did not relate to decreased performance accuracy. There is no evidence, that the individually chosen eye-movement behavior during encoding has any functional relevance. However, even though evidence was generally in favor of the null hypothesis, this evidence was weak for both free versus cued comparisons, but moderate for serial versus cued.

**Table 2.** Summary statistics for correlations between a change in fixation behavior (low-fixation vs. high-fixation probability) and a change in spatial memory performance accuracy in Experiment 2

Presentation	Change between	*r*	*df*	*p*	*R*^2^	BF_10_
Sequential	Free–cued	0.253	28	.178	0.064	0.541
Sequential	Serial–cued	0.127	28	.503	0.016	0.281
Simultaneous	Free–cued	0.236	28	.209	0.056	0.482

*Note.* The analyses deals with the question: Is a change in fixation probabilities between two conditions (low vs. high) related to a change in memory performance? For instance, in case of fixational interference, a strong individual change from low-fixation to high-fixation probability might be related to a loss in memory accuracy. Included is spatial task performance only, due to the fact that fixation probabilities were at the ceiling during verbal encoding. Results indicate null effects in the weak to moderate evidence range. We report the correlation coefficient *r, df*, *p*, *R*^2^, and Bayes factor (BF10)

One might argue that the reported analyzes have the disadvantage of arguing with null effects. However, combining a replication of null effects with a replication of regression benefits should prove them to be of importance.

#### Regressions and the relation to spatial memory encoding

##### Regressions probabilities

We argue in Experiment 1 that regressions have functional relevance to maintain spatial (order) memory. Then, they should occur more often in spatial serial and free recall (serial order memory played a role) than in cued recall (serial order memory played no role). In spatial serial and free recall, they again should be directed mainly to the first list item’s position. And finally, they might benefit accuracy for list items that were regression targets, since Bayesian analysis was generally in favor of such a benefit in Experiment 1.

**Fig. 7:**
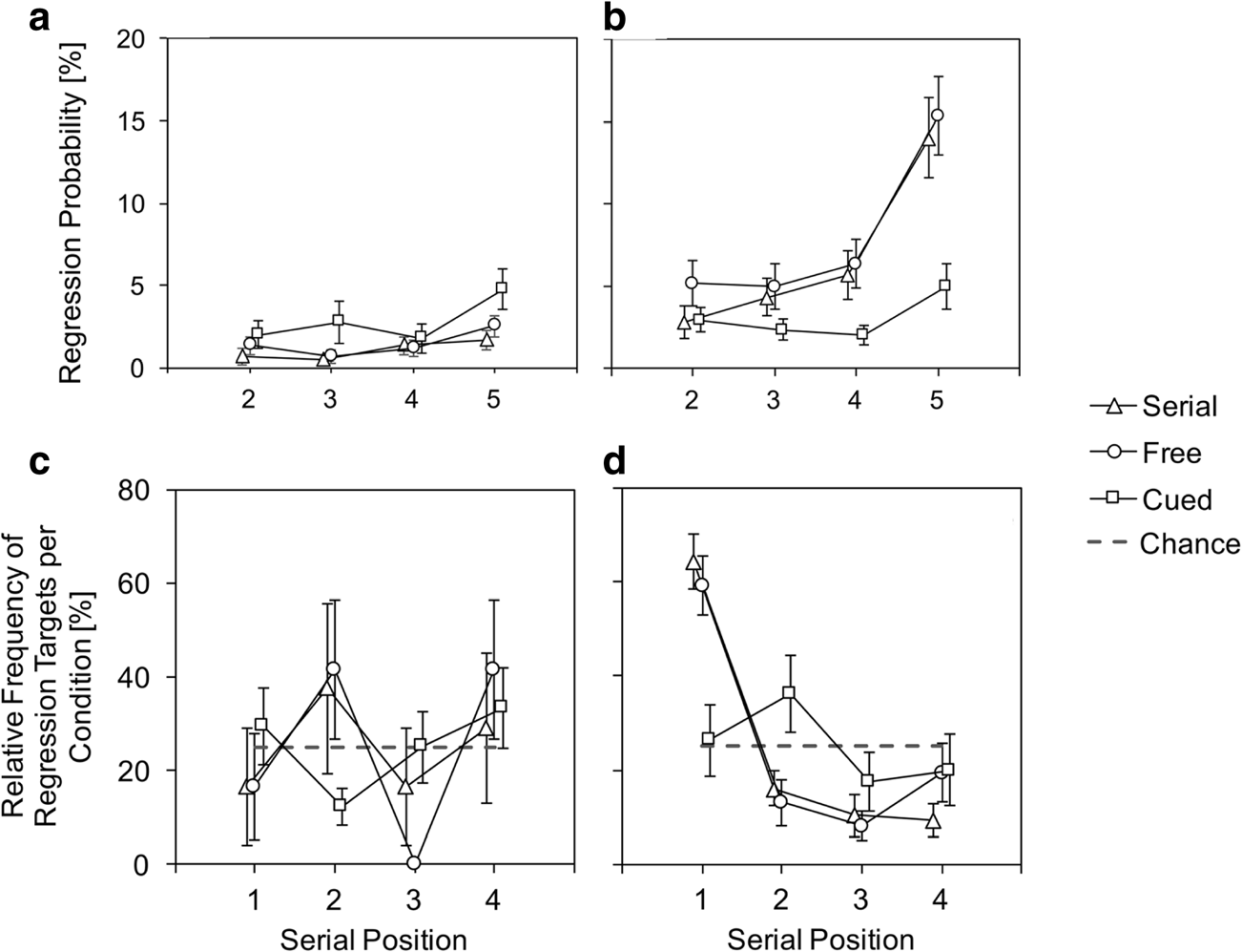
**a–b**, Regression probabilities during verbal (**a**) and spatial (**b**) memory encoding with sequential presentation. Regressions were rare for the verbal tasks as well as for cued spatial recall in which serial order memory did not aid report. Serial and free spatial recall showed a marked preference for regressions during the presentation of the final list item. **c–d** Regression targets analogue Experiment 1 for verbal (**c**) and spatial (**d**) encoding; (**c**) In line with the low-regression probabilities in the verbal tasks (**a**), the distribution of regression targets for the verbal task showed no systematic pattern but corresponded to the chance model. For the spatial task (**d**), the regression distributions clearly deviated from the chance model for serial and free recall, but not for cued recall (we present data for sequential presentation condition only; refixations during simultaneous presentation do not clearly separate encoding from maintenance and were not analyzed). Error bars depict between-subjects standard errors

Indeed, Fig. 7b show higher regression probabilities for spatial serial and free than for cued recall. They appeared to be most prevalent on the last serial position (see Fig. 7b) and again targeted preferentially at the first item position (see Fig. 7d). All other conditions showed a rather fuzzy pattern of regression targets (see Fig. 7c–d). Regressions were overall more prevalent in spatial than in verbal encoding (see Fig. 7a–b). Results are in line with the assumption that regressions play a specific role for spatial maintenance in tasks requiring serial order memory. To foreshadow results, we showed a clear beneficial effect for regressions for spatial serial as well as free recall.

ANOVAs statistically supported our interpretation of Fig. 7. The ANOVA for regression probabilities in the spatial task (see Fig. 7 b) showed a significant main effect of task condition (serial, free, cued), *F*(2, 58) = 6.87, *p* = .002, η^2^ = .19, and of serial position (2 to 5), *F*(1.81, 52.59) = 21.18, *p* < .001, η^2^ = .42 (Greenhouse–Geisser corrected), and a significant interaction, *F*(3.20, 92.87) = 7.71, *p* < .001, η^2^ = .21 (Greenhouse–Geisser corrected). Accordingly, the best model included both factors and the interaction (BF_10_ = 8.47 × 10^16^). The interaction was driven by the deviant profile of regressions in cued recall. Regression probabilities of serial and free recall did not differ, *F*(1, 29) = 1.46, *p* = .237, η^2^ = .05, and showed no interaction with serial position, *F*(1.98, 57.48) = 0.31, *p* = .818, η^2^ = .01 (Greenhouse–Geisser corrected). Accordingly, the best model included only the factor serial position (BF_10_ = 3.96 × 10^12^).

Regression probabilities for verbal recall (see Fig. 7a) did not differ between task conditions, *F*(1.18, 34.19) = 2.43, *p* = .124, η^2^ = .08 (Greenhouse–Geisser corrected). There was a main effect of serial position, *F*(3, 87) = 6.37, *p* < .001, η^2^ = .18, and no interaction, *F*(3.58, 103.77) = 1.57, *p* = .195, η^2^ = .05 (Greenhouse–Geisser corrected). The best model, however, included both the factors task condition and serial position (BF_10_ = 62.84). Post hoc *t* tests of serial position in the verbal task revealed that serial positions 2, 3, and 4 differed significantly from serial position 5 (all *p*s < .006, all BF_10_s > 7.96). There was no significant difference in regression probability between serial positions 2, 3, and 4 (all *p*s > .731, all BF_10_s < 0.21). That is, similar to spatial encoding, regressions in the verbal task were mostly prevalent during presentation of the last list item.

To test for significant differences of regression target positions (see Fig. 7c–d), we calculated one-factorial repeated-measures ANOVAs for each condition. The likelihood of being a regression target differed significantly between the serial positions in spatial free recall, *F*(2.28, 50.24) = 14.28, *p* < .001, η^2^ = .39, BF_10_ = 8.11 × 10^6^ (Greenhouse–Geisser corrected). Similarly, serial position was significant for spatial serial recall, *F*(3, 66) = 26.12, *p* < .001, η^2^ = .54, BF_10_ = 9.62 × 10^11^, respectively. None of the other conditions showed significant deviations of regression target probabilities across serial positions (all *p*s > .10, all BF_10_s between 0.23 and 1.62).

##### Relation between regressions and performance accuracy

We replicated the difference in regression probabilities between verbal and spatial encoding from Experiment 1 for serial as well as free recall. Can we now also replicate the functional role for regressions during spatial encoding? We decided to include the regression analyses for the sequential conditions only, as refixations in the simultaneous condition is qualitatively different (e.g., no longer memory based), and overt encoding and maintenance overlaps. In addition, analysis on cued recall was omitted, as there were almost no regressions during encoding.

Importantly, the performance benefit emerged clearly, much stronger than in Experiment 1, in serial as well as free spatial recall. Recall accuracy in serial recall was higher for regression targets (*M* = 83.09%, *SD* = 24.02) than for items that were no regression targets (*M* = 67.58%, *SD* = 20.45), *t*(26) = 3.19, *p* = .004, BF_10_ = 10.91. Likewise, free recall accuracy for regression targets (*M* = 88.43%, *SD* = 16.49) was higher than for nonregression targets (*M* = 79.91%, *SD* = 12.53), *t*(26) = 2.67, *p* = .013, BF_10_= 3.76. That is, we replicated the beneficial role of regressions for memory performance accuracy of the regression targets.

#### Individual differences in oculomotor behavior during spatial memory encoding

**Fig. 8:**
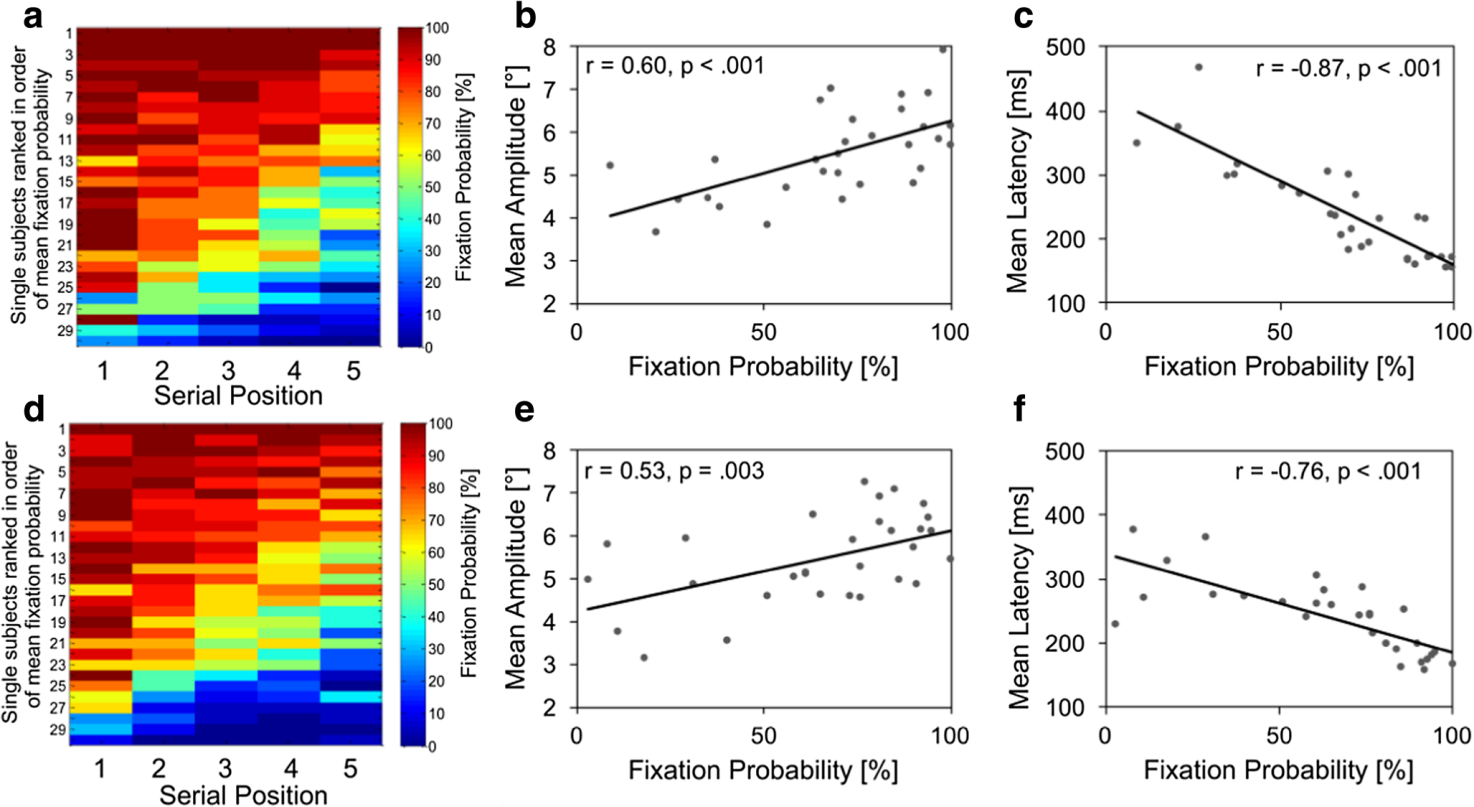
Individual differences in eye movement behavior during encoding of spatial serial (**a–c**) and free recall (**d–f**) with sequential presentation. From left to right, subplots depict individual differences in fixation probabilities on serial position (**a, d**), the correlation between individual mean fixation probability and saccadic amplitude (**b, e**), and the correlation between individual mean fixation probability and saccadic latency (**c, f**). For sequential presentation, latencies for the first saccade after item onset are analyzed. All correlations are BF > 17

Figure 8 depicts individual differences in oculomotor behavior for spatial serial and free recall with sequential presentation. We spared analysis for cued recall in sequential presentation, as probabilities were at the ceiling and did not offer variance. For simultaneous presentation, there is no equivalence for saccadic latencies, which are related to the onset of each item presentation in sequential presentation.

Similar to Experiment 1, the fixation probability profile across serial positions varied strongly between participants (e.g., some showed high-fixation probabilities across all serial positions, some low). Plotting individual mean fixation probabilities by saccadic amplitude or by saccadic latencies revealed linear relations and high correlations. The systematics of relations converge to the conclusion of individual differences in saccadic suppression. Tables 1 and 2 reported that there was no systematic relation between eye-movement strategy and accuracy. The conclusion is that interindividual differences in fixation patterns exist, but there is no advantage for one specific strategy in comparison with the others.

#### How free is free recall?

**Fig. 9:**
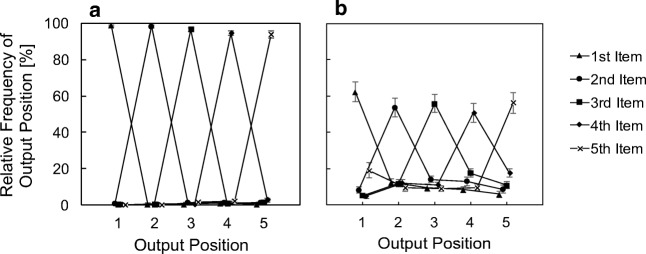
Relative frequency of a specific output (recall) position given a specific input (presentation) position for the free recall task in Experiment 2. **a** Verbal free recall. **b** Spatial free recall. Relative frequencies of about 1 means that the serial input position matched the serial output position. This was particularly prevalent in verbal free recall and particularly for the early list positions. However, even in spatial free recall order was largely maintained. Error bars depict between-subjects standard errors

We collected data for serial and free recall during one and the same experimental session (task order balanced between participants). In this context, participants might have adopted a serial memory strategy for free recall. One might be skeptical on the validity of the claim, we replicated the effects in a different task design than serial recall. Figure 9 presents the relative frequency of output position given a specific input position. In the verbal task, serial output closely matched input. Matching input–output positions (the peaks in Fig. 9) occurred in more than 95% cases. Memorization of items in serial order guided verbal free recall, making verbal serial and free recall indistinguishable in terms of serial-order requirements. In contrast, matching positions in the spatial task occurred on a level of about 60%. Even though serial order played a role in spatial free recall as well, this role was much less pronounced than in spatial serial recall. Despite those differences in recall order between spatial serial and free recall, fixation probabilities did not differ between spatial free and serial recall (see Fig. 6, sequential presentation), neither did regression probabilities (see Fig. 7). The conclusion is: The suppression effect of fixation probabilities and increased regressions in spatial memory are not related to the requirement of serial-order memory.

### Discussion

We compared eye-movement behavior for serial, free, and cued recall in Experiment 2. We removed the affordance to encode serial order information to understand, whether our effects from Experiment 1 were specific for spatial serial memory or transfer to other types of spatial memory encoding as well. In addition, we added another task than that in Experiment 1 to induce a change in fixation probabilities (here: cued recall). The suppression effect (reduced fixation probabilities for spatial in comparison with verbal) replicated, did not differ between serial and free recall, and showed for simultaneous as well as sequential presentation in the free recall task. In addition, replicating Experiment 1 again, saccadic suppression did not relate to memory performance, and the task-induced change from low-fixation to high-fixation probabilities had no consequence as well. In contrast to saccadic suppression, the regressions were functional for memory accuracy in serial as well as free recall. Regressions showed mainly for spatial memory tasks, indicating their domain-specific benefit. Again, results replicate Experiment 1. Finally, fixation behavior (serial and free recall, sequential presentation) was individually different, highly systematic, with lower fixation probabilities related to lower saccadic amplitudes and higher saccade latencies, but no advantage of a specific eye-movement strategy showed, replicating Experiment 1 as well.

## General discussion

In two experiments, we studied eye-movement behavior during encoding of a number of verbal items or their spatial location in memory tasks. We were primarily interested in understanding the role of eye movements for serial spatial encoding that requires to maintain past information while concurrently integrate upcoming input. There were a number of important findings: We demonstrated that fixation probabilities for on-screen items were high in the verbal task, but much decreased in the spatial task; this decrement was not related to memory performance. Although there was a broad range of spatial encoding strategies in our sample, from complete overt to complete covert encoding of spatial locations, differences in encoding strategy did not drive differences in memory performance. In addition, a task-induced change in encoding strategy (from covert to overt) did not affect memory performance. In contrast, regressions to locations on prior on-screen items predicted improved spatial recall. Importantly, we replicated results across different tasks (serial, free recall) and presentation modes (serial, simultaneous), showing that our findings are reproducible and generalize.

Our results demonstrate a dissociation between saccadic activity and visuospatial working memory. This finding was unexpected, given the repeatedly shown reduced saccadic activity in spatial memory tasks (Experiments 1 & 2; Lange & Engbert, 2013; Patt et al., 2014) and the fact that interference between the eye-movement system and visuospatial working memory has been demonstrated in a variety of studies and task designs (e.g., Belopolsky & Theeuwes, 2009b; Hale et al., 1996; Lange et al., 2012; Lawrence et al., 2001; Pearson et al., 2014; Postle et al., 2006; Theeuwes et al., 2006). In our studies, saccade frequencies did not differ between the verbal and spatial task. But saccades in the spatial task were often not directed to the to-be-encoded locations and showed prolonged latencies.

On theoretical grounds, it is very plausible to assume that saccades interfere with spatial memory representations due to remapping processes. As spatial relations are represented in a retinotopic way (e.g., Engel, Glover, & Wandell, 1997), the requirements to transform retinotopic coordinates into a world-fixed spatiotopic frame (Golomb, Chun, & Mazer, 2008) introduce noise into spatial memory representations. For example, retinotopic position recall is superior to spatiotopic (Vasquez & Danckert, 2008), and saccadic interference is accumulative for spatiotopic but not retinotopic memory with each additional saccade (Golomb & Kanwisher, 2012). Behavioral evidence of saccade errors converges with the hypothesis of remapping costs: The representation of a memorized location shifts in relation to an irrelevant saccade (Henriques, Klier, Smith, Lowy, & Crawford, 1998), and this shift is related to what can be expected from location remapping. Lastly, there is another challenge for the oculomotor system: The facilitation of perceptual processing at an attended location is followed by inhibition (Klein, 2000). This inhibition of return (IOR) was shown by covert attention shifts but also for saccadic movements. That is, making saccades to specific locations subsequently inhibits attention allocation to these locations. In this respect, making a saccade enhances perceptual processing at this location but might hinder further processes related to shifting attention to this location. It has been demonstrated that maintenance of spatial memory representations affects the saccade system (e.g., saccade trajectories deviate from the memorized position, indicating inhibition), but it does not affect the IOR effect (Theeuwes et al., 2006; Zhang & Zhang, 2011). That is, inhibition by memory maintenance and by IOR can be differentiated. The results of our study demonstrate that our cognitive system is able to deal with affordances like remapping and inhibitory processes under free (i.e., natural) viewing behavior.

Even though we were surprised by our dissociation of saccadic activity and visuospatial working memory, evidence is highly compatible with our findings. For example, a direct test of saccadic costs has been conducted with a location memory task, in which recall was cued by the color of the to-be-remembered object, as well as with an orientation task for objects at specific locations, again cued by color (Bays & Husain, 2008). Both tasks require memory for different locations and bindings of representations for location and other object features (e.g., color). Making a saccade toward one object before recognition of location or orientation did not have a negative effect on task performance (e.g., comparing an interleaved saccade before recognition with a fixation condition). In contrast, precision for location and orientation memory increased for a saccade target and decreased for nontargets. When more than one object was fixated sequentially, precision was highest for the last saccade target with a marked drop of precision for all other earlier saccade targets. This leads to two important interpretations: resources for saccade execution, remapping, and transsaccadic memory are different from visuospatial memory for bound objects, and resources are shifted flexibly and quickly from one saccade target to the next, with only the last saccade target showing increased precision. The first interpretation is also supported by a study on visual perception that differentiated between resources needed for transsaccadic memory and intentionally encoded visuospatial information (Poth & Schneider, 2018). Increasing transsaccadic memory load (by an increased number of digit objects placed near the target of a goal-directed saccade) did not affect visual perception, but increasing short-term memory load did (memorize the digits for a later recognition task; Poth & Schneider, 2018). Accordingly, in our study, saccades to to-be-encoded locations during presentation were neither functional nor created interference. They simply played no role for later memory performance. 

There seems to be one exception: The last saccade target in a series has a more vivid trace in memory (Bays & Husain, 2008; see also Körner & Gilchrist, 2007; for a theoretical account, see Niklaus, Singmann, & Oberauer, 2019). This might fit to our regression effects: Regressions were mostly placed during presentation of the last item and targeted the very first item. Rehearsal in verbal memory is particularly directed to the list beginning (Tan & Ward, 2008). In addition, saccades in the retention interval are beneficial when placed onto the first three to-be-recalled locations (Godijn & Theeuwes, 2012). Those results converge with working memory models covering serial-order memory, which assign importance to the encoding of the beginning of the list (e.g., Brown et al., 2007; Henson, 1998; Lewandowsky & Murdoch, 1989; Page & Norris, 1998). Our findings on regressions fit nicely to this literature. Regressions might boost memory for the first item. Proper retrieval of the first position in the series might cue subsequent items (e.g., by chaining), supporting serial order memory.

An overwhelming amount of studies show overlap between the oculomotor system and visuospatial memory. Our findings do not speak against this relation. Task designs might play an important role for the outcome and the oculomotor system adapts to task affordances. We used an observational account with free viewing instructions. The missing effect of on-item fixations on memory performances demonstrate the high efficiency of the oculomotor system in this setting. This points to another challenge in this research field: For better control, researchers often limit task designs to memory for one item (e.g., Awh, Jonides, & Reuter-Lorenz, 1998; Belopolsky & Theeuwes, 2009a, 2009b; Boon, Belopolsky, & Theeuwes, 2016; Henriques et al., 1998; Hollingworth & Luck, 2009; Sprague & Serences, 2013). The finding of strong overlap between the oculomotor system and visuospatial memory might relate to this fact. During a sequence of saccades, the oculomotor system might attribute resources in a more flexible way (Bays & Husain, 2008), and relations might become less traceable. This conclusion is also reported by comparing neural activity maps for location memory of one in comparison with two items (Sprague, Ester, & Serences, 2014). Neural item representations of two items are not disjointed, which is related to less precise memory performance. A neural priority map codes importance within the visual field and can be found across different hierarchical levels in the visual system (e.g., Sprague & Serences, 2013). Priority maps have been demonstrated for maintenance in working memory, covert visuospatial attention, and saccade goals (e.g., Bisley & Goldberg, 2010; Duhamel, Colby, & Goldberg, 1992; Fecteau & Munoz, 2006; Jerde, Merriam, Riggall, Hedges, & Curtis, 2012; Sommer & Wurtz, 2001; Umeno & Goldberg, 2001). The demonstrated overlap between the oculomotor system and visuospatial memory has been interpreted as independent modules that share a common priority map (Boon, Belopolsky, & Theeuwes, 2016) or that incorporate priority maps that are highly interrelated (Theeuwes, Belopolsky, & Olivers, 2009). Given the importance of serial recall within the memory literature, it seems a crucial next step to develop models to incorporate results from tasks including more than one memory location.

What do our results tell us about spatial memory representations? There is evidence that at least two different codes are involved in spatial memory (Lecerf & de Ribaupierre, 2005; Pazzaglia, 1999; Ridgeway, 2006): path encoding and pattern encoding. Both represent relations between individual items. Path encoding traces the relations in a sequence. Movements from one position to the other can be part of path encoding. This code corresponds to route descriptions in mental imagery. Pattern encoding creates a more holistic representation. Individual items are grouped into ensembles, like the spatial layout of a map instead of a route. Evidence for path encoding comes from effects of path complexity, which results from differences in path length, number of path crossings, and angle sizes at turn-offs (Busch, Farrell, Lisdahl-Medina, & Krikorian, 2005; Orsini, Pasquadibisceglie, Picone, & Tortora, 2001; Parmentier & Andrés, 2006; Parmentier, Elford, & Maybery, 2005). With higher path complexity, spatial serial memory decreases. Evidence for pattern encoding in spatial serial recall has been demonstrated by effects of pattern symmetry, repetitions of translated subgroup positions, and gestalt principles like continuation (Kemps, 2001; Rossi-Arnaud, Pieroni, & Baddeley, 2006; Rossi-Arnaud, Pieroni, Spataro, & Baddeley, 2012). It has been suggested that eye movements might particularly support path encoding (Guérard et al., 2009). However, we showed that fixations onto to-be-remembered items are not functional, contrasting the crucial role of eye movements for path encoding. Is the suppression effect then indicative of pattern encoding? Unfortunately, our study does not contribute to this question. We did not experimentally differentiate between spatial codes. It might very well be that reduced oculomotor activity accompanies or even promotes pattern encoding. For example, reduced activity might enhance retinotopic encoding of a pattern and support binding of single locations into one pattern (e.g., Schneider, 1999). When participants were forced to switch from low-fixation probabilities during spatial encoding to high-fixation probabilities during combined encoding, they might have changed spatial codes from pattern to path encoding. However, memory performance was not affected by the induced change of fixation probabilities and the potential change of coding. We have no measure for the type of code applied by individual participants in individual trials. It is then outside the scope of or study to relate eye-movement behavior to a specific spatial code. Sophisticated designs are necessary to understand the interplay between eye movements and specific types of spatial encoding.

Further studies should also manipulate set size, as sequences within or exceeding individual span will require different processes. This might correspond to the finding that fixation probabilities in spatial recall decreased with increased serial position. In addition, varying presentation times from very short (e.g., 250 ms) to very long (e.g., 4 s) would uncover dynamic changes in encoding strategies. The longer the presentation times, the more time will be dedicated to maintenance processes. Whereas short presentation times limit the possibilities for eye movements to take place, long presentation times allow for complex fixation sequences. It would be quite interesting to see whether saccadic suppression remains for long presentation times as well, and if not, when and why a suppression strategy might change to a fixation strategy.

Another interesting point for further studies is to incorporate other motor systems in spatial memory tasks. For instance, pointing movements and movement preparations have been demonstrated to interact with visuospatial memory representations (e.g., Hale et al., 1996; Lawrence et al., 2001; Rossi-Arnaud, Longobardi, & Spataro, 2017; Spiegel, Koester, & Schack, 2014). The model of shared priority maps (Hedge, Oberauer, & Leonards, 2015; Theeuwes et al., 2009) seems to be compatible with this evidence. Interestingly, pointing as intentional action has been argued to enforce selective attention and facilitate the generation of spatial representations (Chum, Bekkering, Dodd, & Pratt, 2007). The additional motor code might particularly benefit spatial serial recall (Dodd & Shumborski, 2009). However, findings on intentional pointing to spatial positions in the context of spatial memory show mixed results. Whereas one study clearly shows benefits for pointing in comparison with passive viewing (Chum et al., 2007), others show memory impairment in the pointing condition (Spataro, Marques, Longobardi, & Rossi-Arnaud, 2015). Benefits and impairments by pointing seems to be highly dependent on task procedure (e.g., blocked condition versus manipulation within trials; Dodd & Shumborski, 2009) and on specific serial positions (Rossi-Arnaud et al., 2017). Often, particularly the first serial positions suffer from pointing (Rossi-Arnaud et al., 2017). This is opposite to our finding of regression benefits to the first serial position. However, pointing studies investigate intentional motor performance, but in our study, eye-movement measures were spontaneous.

We interpret our findings in the framework of spatial working memory. However, our stimuli were letters presented visually at specific positions. This task taps not only into spatial but also into memory for visual features. It is worth noting that the saccade system is also involved in selecting visual information into short-term or working memory. For instance, saccades to positions of orientation stimuli, which were no longer visible, increased visual memory performance (Ohl & Rolfs, 2017). Executed or planned saccades to placeholders in a retention interval mediates memory for object features, encoded earlier on this position (Hanning, Jonikaitis, Deubel, & Szinte, 2015). Such saccade-target benefits might depend on the time for visual processing of the target (Ohl & Rolfs, 2017), because there is no such saccade target benefit in a visual discrimination task in which peripheral, visual stimuli were presented very briefly (100 ms) followed by a mask (Khan, Blohm, Pisella, & Munoz, 2015). In this discrimination task, covert attention, but not saccadic planning or execution towards the target location, increased performance. Besides gating into visuospatial short-term memory, spatial attention has been shown to play a major role in binding tation (Clark, Noudoost, & Moore, 2012) and to benefit memory representations for visual objects located at the attended location (Griffin & Nobre, 2003; Landman, Spekreijse, & Lamme, 2003) That is, even in the absence of a stimulus, spatial location can cue visual stimulus properties (see also, the “looking at nothing” paradigm; Ferreira et al., 2008; Richardson & Spivey, 2000). We were interested in the relation between natural eye-movement behavior and spatial memory encoding in a spatial serial recall task. This task also involves visual information (e.g., letters) as well a binding of visual-verbal and spatial information (combined condition in Experiment 1 and cued recall in Experiment 2). We cannot draw any firm conclusion on the role of eye movements regarding these two aspects. In our design, however, saccadic suppression occurred for the spatial memory tasks only, not for combined object features, indicating different eye-movement strategies in these tasks.

We explored eye movement behavior during memory encoding of visually presented stimuli that can be encoded into the spatial or the verbal memory domain. In doing so, we seek to illustrate and understand domain-specific eye-movement control by showing dissociations between verbal and visuospatial working memory. Dissociations between verbal and visual spatial working memory have been demonstrated by an overwhelming amount of evidence from dual-task studies, patient studies, and neuroimaging (Baddeley, 1986; Baddeley, Grant, Wight, & Thomson, 1975; Brooks, 1967, 1968; Bruyer & Scailquin, 1998; Logie, Zucco, & Baddeley, 1990; McConnell & Quinn, 1996, 2000; Morris, 1987; Quinn & McConnell, 1996; E. E. Smith & Jonides, 1999; for an overview, see Baddeley, 2003; Hurlstone, Hitch, & Baddeley, 2014). Applying such a task design revealed the interesting observation of saccadic suppression in the spatial, but no such suppression in the verbal task. Moreover, it demonstrated the functional role of regressions for spatial but not verbal memory encoding. We showed, then, that spontaneous eye-movement control is different between encoding into different task domains.

In our study, we decided on an observational account. Overt behaviors are valuable cues to understand, how humans engage with a task and deal with task affordances (Morey et al., 2017). We decided against the experimental manipulation of viewing behavior (e.g., forced viewing instructions) for two reasons: First, evidence showed decreased memory performance independent of task domain (verbal or spatial; Lange & Engbert, 2013) when forced viewing was added. This strongly suggests dual-task costs for eye-movement control by the additional viewing task. It corresponds to research on cross-modal attention, which reports growing evidence for the oculomotor system to create dual-task costs (for a review, see Huestegge, 2011). Dual-task costs are problematic because it is difficult to differentiate these from other interference effects. Second, given the demonstrated dual-task costs of forced viewing instructions, it is likely that participants try to find specific strategies to handle these task affordances. Hence, designs with forced viewing instructions likely include measures of instruction-adaptive or design-adaptive solution strategies, which are difficult to differentiate from the measure in question (e.g., natural eye-movement control). The observational account allowed us to investigate natural eye-movement behavior during memory encoding and revealed a surprising dissociation between oculomotoric activity and spatial memory.

## Electronic supplementary material

ESM 1 (DOCX 4.19 MB)

## Footnotes

1 For the purpose of our study, we will term saccades for potential maintenance regressions. These are saccades to item locations that have been presented but are no longer visible on the screen. The terminology is motivated by reading research, where fixations onto locations to text backwards in reading direction are called “regressions.” In our context, fixations are directed to locations that were presented during the past encoding phase and have to be remembered, going backwards in time. Different from reading, memory regressions are thereby often targeting at “empty” locations, where a visual object is no longer presented.

2 The attentive reader might note the higher fixation probability for the verbal task in the current study in comparison with Lange and Engbert (2013), which is probably due to the shorter list length and other differences in the design.
