# Supplementary material for: On-item fixations during serial encoding do not affect spatial working memory
Source: Atten Percept Psychophys. 2019 Jun 28;81(8):2766–87. doi: 10.3758/s13414-019-01786-5 (PMC6856038; doi:10.3758/s13414-019-01786-5)
Supplement: Supplementary file 1 — (DOCX 4.19 MB) [file 13414_2019_1786_MOESM1_ESM.docx]

On-item fixations during serial encoding do not affect spatial working memory

Supplementary material

**Experiment 1**





Figure 1. (A – D, verbal recall) Individual fixation probabilities onto items show consistently high probabilities across serial positions (A). There are no significant correlations between fixation probability and (B) smaller mean saccadic amplitude, (C) higher saccade latencies, or (D) recall accuracy. (E – H, combined recall) As in verbal recall, individual fixation probabilities onto items show consistently high probabilities across serial positions (E). There are no significant correlations between fixation probability and (F) mean saccadic amplitude, or (H) spatial recall accuracy, nor (I) verbal recall accuracy. But (G) higher saccade latencies shows a significant negative correlation.

**Experiment 2**





Figure 2. Individual fixation probabilities onto items show consistently high probabilities across serial positions in spatial cued recall (A), verbal serial recall (D), verbal free recall (G), and verbal cued recall (J). Mean amplitude shows a general trend to correlate negatively with fixation probability (B, E, H, K).
